# Neuroimaging at 7 T: are we ready for clinical transition?

**DOI:** 10.1186/s41747-021-00234-0

**Published:** 2021-08-26

**Authors:** Mirco Cosottini, Luca Roccatagliata

**Affiliations:** 1 Department of Translational Research On New Technologies in Medicine and Surgery, University of Pisa, Pisa, Italy; 2 Department of Health Sciences (DISSAL), University of Genoa, Via Pastore 1, 16132 Genoa, Italy; 3 Department of Neuroradiology, Ospedale Policlinico San Martino IRCCS, Genoa, Italy

**Keywords:** Magnetic resonance imaging, Neuroimaging, Neurodegenerative diseases, Cerebrovascular diseases, Multiple sclerosis

## Abstract

In the last 20 years, ultra-high field (UHF) magnetic resonance imaging (MRI) has become an outstanding research tool for the study of the human brain, with 90 of these scanners installed today, worldwide. The recent clearances from regulatory bodies in the USA and Europe to 7-T clinical systems have set the ground for a transition from pure research applications to research and clinical use of these systems. As today, UFH neuroimaging is demonstrating clinical value and, given the importance of this topic for both preclinical scientists and clinical neuroradiologists, *European Radiology Experimental* is launching a thematic series entitled “7-T neuro MRI: from research to clinic”, consisting of peer-reviewed articles, invited or spontaneously submitted, on topics selected by the guest editors, describing the state of the art of UHF MRI neuroimaging across different pathologies, as well as related clinical applications. In this editorial, we discuss some of the challenges related to the clinical use of 7-T scanners and the strengths and weaknesses of clinical imaging at UHF.

From the installation of the first 7-T magnetic resonance imaging (MRI) system at the University of Minnesota more than 20 years ago [1], ultra-high field (UHF) MRI (*i.e.*, those using a magnet generating a B_0_ field ≥ 7 T) has progressively affirmed its role as a powerful technology for human neuroimaging in several research centres worldwide. As of today, there are approximately 90 scanners for human imaging operating at UHF [2] with growing efforts in the development of next generation UHF magnets for human imaging operating at up to 20 T [3].

In 2017, the United States Food and Drug Administration gave 510(k) clearance to a 7-T clinical system from one manufacturer, which in the same year was also appointed with a *conformité Européenne*−CE mark [4, 5], and in late 2020, another vendor announced Food and Drug Administration clearance for a 7-T scanner [6]. These approvals have set the ground for a transition from pure research applications to research and clinical use of 7-T MRI systems.

Today, UFH neuroimaging is demonstrating promising clinical applications and, given the importance of this topic for both clinical radiologists and preclinical scientists, *European Radiology Experimental* is launching the thematic series entitled “7-T neuro MRI: from research to clinic”. This series consists of peer-reviewed articles, including invited review articles on topics selected by the editors, describing the state of the art of UHF MRI neuroimaging across different pathologies, as well as related clinical applications.

Moving towards UHF brings not only opportunities, but also considerable challenges for clinical imaging. With the increase of the static (B_0_) magnetic field strength, MRI physics determine pros and cons for diagnostic applications [7].

The increase of the magnetic field strength leads to greater spin polarisation in the tissue. The signal increases quadratically with the strength of the static magnetic field while the associated noise demonstrates linear dependence; hence, the use of 7-T systems leads to a significant increase in the signal to noise ratio (SNR). The increased SNR can be exploited to increment the spatial resolution enabling to visualise small anatomical structures (in the order of hundreds of microns) or to increase the temporal resolution of functional studies. The higher sensitivity with increasing B_0_ also motivates the implementation of multinuclear imaging, to allow the study of other nuclei that are less abundant than ^1^H in humans (such as ^13^C, ^23^Na, ^31^P).

The major benefits of 7-T imaging are observed in brain studies, especially for those techniques exploiting magnetic susceptibility phenomena, such as *susceptibility-weighted imaging* (SWI) and functional MRI (fMRI). Since the magnetic susceptibility effects scale linearly with B_0_, 7-T MRI enables new types of contrasts based on the small difference in susceptibility that are not unveiled at conventional magnetic field strength. The higher sensitivity to susceptibility has introduced a new frontier for SWI, which is able to create contrasts between tissues containing different amount of paramagnetic o diamagnetic substances such as iron, deoxyhemoglobin, hemosiderin, myelin, or calcium. SWI at UHF enables high-resolution imaging with unprecedented anatomical detail and related techniques such as *quantitative susceptibility mapping*−QSM can supply information about microstructure and composition of brain tissue. Additionally, the higher sensitivity to deoxyhemoglobin, and thus to the *blood oxygenation level dependent*−BOLD effect, improves the detection of the neurovascular coupling at the basis of fMRI, opening the perspective to boost the exploration of functional activation even at the level of single subjects with higher spatiotemporal resolution and sensitivity [1].

Increased spatial/anatomical resolution fosters new possibilities for the functional study of small structures including cortical columns and laminae [8] and subnuclei of the brainstem [9]. The side effect, however, is linked to the signal loss associated with susceptibility-induced distortions and inhomogeneities of the static magnetic field B_0_ that are more evident at bone air interfaces, for instance at the cranial base, and are a drawback of whole brain imaging, limiting some clinical applications.

A further diagnostic gain could derive from the changes in relaxation times. An example is the improvement in magnetic resonance angiography (MRA) with *time-of-flight*−TOF techniques favoured by the increased longitudinal relaxation time T1 at 7 T promoting the background suppression and enhancing the vessel flow related signal. In fact, at 7 T, the background stationary spins have a longer T1 and they better saturate in the time between the radiofrequency (RF) pulses of the time-of-flight sequence, increasing the contrast of the flowing spins within the peripheral small vessels.

Research and technological advancements are now focused on supporting the gains of UHF and solving potential issues related to the application of UHF MRI to humans. The realisation of more powerful gradient coils with greater amplitudes and slew rates is a prerequisite for whole brain imaging with further increased spatial resolution and reduced geometric distortions. Amelioration of gradients performance would favour, for example, the application of *echo-planar*−EPI for DWI and fMRI (see the paper by Vachha et al. in this series [10]). Higher order shimming gradients aim to improve the homogeneity of the static magnetic field to fully exploit the UHF potential in improving the spectral resolution of magnetic resonance spectroscopy−MRS.

The parallel transmission of the RF with multiple independent channels allows to modify the RF (amplitude and phase) separately for each transmission channel to limit the B_1_ inhomogeneity within the brain due to the dielectric effect [11]. The development of multiple channel receiver coils with smaller and denser detectors for parallel imaging at UHF further enabled to increase the SNR and reduce the acquisition time [12].

The anatomic regions exposed to electromagnetic fields at the increased resonance frequencies at UHF experience a temperature increase correlated with the mean *specific absorption rate*−SAR. At UHF, the dielectric effect induces an inhomogeneous distribution of energy with possible hot spots within an organ. For safety reasons [13], the specific absorption rate measurement has been particularly investigated to obtain a local estimation [14] and reduction [15].

The translation of UHF to clinical applications implies the absence of dangerous side effects and the demonstration of a diagnostic gain. Since the introduction of UHF in the research environment, tens of thousands of MRI examinations have been performed on humans without reporting additional serious adverse side effects with respect to conventional clinical systems. Although discomfort seems to increase with the intensity of magnetic field strength, MRI exams at 7 T appear to be well tolerated by most subjects [16].

On the other hand, at 7 T, the shortening of the RF wavelength can interfere more strongly with metal objects and induce heating of the tissue. Metal implants remain a contraindication to UHF MRI potentially limiting widespread clinical use of UHF although published data report only minor temperature changes in some small implants [17, 18].

Concerning the diagnostic gain, numerous research articles demonstrate the clinical advantage of using 7-T systems in diagnosing central nervous system diseases, also in comparative studies with conventional MRI systems. There are several areas of interest in which 7-T imaging has been tested and we believe that some might be particularly promising in the clinical arena.

Multiple sclerosis (MS), the most common immune-mediated inflammatory demyelinating central nervous system disorder, has been extensively evaluated, and in this series, Bruschi et al. [19] provide an extensive review on the applications of UHF 7-T MRI to the study of both MS and other demyelinating diseases. The use of UHF MRI has become an important technology to investigate central nervous system involvement in MS and has narrowed the gap between the macroscopic view of the radiologist and the microscopic view of the pathologist, for instance identifying *in vivo* the paramagnetic rim sign that may be a marker of compartamentalised inflammation at the lesion edge [20]. The use of 7-T MRI also improves the detection of cortical plaques in MS, in particular of those in subpial location. These lesions are not usually evaluated when imaging patients at 1.5 T and 3 T MRI, but it is well known that this type of cortical pathology contributes to neurological disability and, although more prominent in secondary progressive MS, it is present from the earliest disease phases. Thus, cortical lesions detection might have a role in patient monitoring with appropriate protocols [21]. Furthermore, 7-T MRI has been demonstrated to be specific in revealing the perivenular distribution of MS plaques and the identification of a venule at the centre of MS lesions, the so called central vein sign. This sign has received great interest for its clinical contribution in the differential diagnosis of white matter pathologies and for its potential in increasing confidence in the radiological diagnosis of MS [22].

Another area of interest of 7-T MRI research is epilepsy. In studying drug-resistant epilepsy, 7-T MRI has demonstrated added diagnostic value in revealing epileptogenic lesions. At 1.5-T or 3-T magnetic field strengths, approximately 60−85% of MRI examinations reveal such lesions. Studies have demonstrated that in patients with drug-resistant epilepsy, review of 7-T MR images can unveil lesions which are undetected on images obtained at lower fields [23]. Additionally, it has been recently proposed that, with appropriate protocols, 7-T MRI would be particularly useful in clinical settings for presurgical evaluation or for better epileptogenic lesions identification and classification [24].

In the evaluation of neurodegenerative disorders (see the article of Düzel et al. in the series [25]), UHF MRI provides new radiological markers of disease in pathologies with unremarkable conventional MRI examinations at lower field strength.

In Parkinson disease, 7-T MR has been used to visualise alterations within the substantia nigra [26], resulting in loss of the normal appearance of its dorsolateral area, which is accepted as a radiological sign of nigral pathology, useful to increase the diagnostic accuracy compared to conventional MRI systems [27].

With its superior imaging contrast and resolution, 7-T imaging provides an improved visualisation of *deep brain stimulation*−DBS target nuclei in Parkinson disease compared to conventional 1.5-T or 3-T clinical scanners [28, 29].

Additionally, SWI at 7 T allows the visualisation of cortical laminar structure, enabling to detect atrophy and signal hypointensity in the deep layers of the primary motor cortex of patients with *amyotrophic lateral sclerosis*−ALS [30].

Submillimetre spatial resolution of anatomical T1-weighted images obtained with 7-T MRI has been shown to enable the estimation of cortical thickness through the reduction of partial volume effects and better segmentation of the hippocampal subfields and amygdalar nuclei [31, 32]. These measures can be used as markers of neurodegeneration in *Alzheimer disease*−AD, even in the presymptomatic phase of the disorder with the purpose to monitor emerging disease modifying therapies.

In cerebrovascular diseases, UHF MRI demonstrates its value in improving the identification and characterisation of different types of pathology, including microbleeds, leveraging on its superiority in SWI [33] and ischemic lesions which are often invisible at lower MRI fields such as cortical microinfarcts [34]. Another emerging field of application of UHF MRI is the imaging of intracranial arteries anatomy and pathology: high-resolution (MRA) allows improved detection of small arterial vessel such as the lenticulostriate arteries [35]. Assessment of intracranial atherosclerosis favours 7-T MRI with respect to 3-T MRI, with greater vessel wall visibility and more lesions detected [36]; UHF MRI opens new frontiers in the imaging of intracranial aneurysms thanks to the identification of aneurysm wall microstructures not depictable at lower spatial resolutions [37]. The combination of MRI and MRA techniques thus enables the assessment of various aspects of cerebrovascular disease at the level of both brain parenchyma and cerebral vasculature, allowing the visualisation of pathological features that are often unrecognised at lower MRI fields [38, 39].

The articles published in these thematic series should intrigue the readers of *European Radiology Experimental* who are interested in the debate rising within the radiologist’s community about the 7-T MRI transition to a clinical setting. Some of the aspects of the debate on the future of clinical UHF applications seem to replicate the discussion of the first decade of the century about the implementation of 3T in the clinical work-up.

Even if 1.5-T systems are still the most used scanners, 3-T clinical systems are today commonly present in neuroradiology departments, but in the early 2000s, some radiologists opposed the clinical use of 3 T, given the burden of artefacts that were complex to overcome and often required the shift towards unfamiliar pulse sequences to achieve better image quality [40]. On the other hand, other radiologists were already appreciating strengths over limitations when using second generation 3-T scanners equipped with multichannel receiver coils and with parallel imaging capabilities [41, 42].

The advantages of UHF imaging in neuroscience research are clear and enormous. Without any doubt, there are great potentials for clinical imaging, with applications that can benefit from 7-T MRI that are becoming well defined. Nevertheless, the high costs and the complexity in operating these systems might impede their fast widespread installation and one possible realistic scenario for the next future is that their installation will be confined to a relatively limited number of radiological hubs where UHF studies would be obtained as a complementary examination to 1.5-T and 3-T studies to provide additional information not achievable at lower fields.

Today, there are mixed expectations with enthusiastic proponents of the clinical use of 7-T MRI on the one side and clinicians showing a more conservative approach on the other. The cautious arguments derive from considerations on high costs for installation, more complex maintenance and calibration, need for revision and fine tuning of acquisition protocols, and completion of the technical refinements for whole brain imaging acquisitions. Moreover, the clinicians reading UHF MRI studies will have to become acquainted with unprecedented contrasts and higher anatomical resolution and they will have to acquire new skills for the correct interpretation of these outstanding images.

What we know and also this thematic series shows is that in neuroscience, UHF MRI can significantly improve the clinical diagnostic process in selected pathologies. With time, we will know if this gain will be sufficient to motivate a relatively widespread installation of 7-T scanners in hospitals for clinical use. If yes, it will be again a new scale for MRI, again starting from neuroimaging.

## Data Availability

Not applicable.

## Abbreviations

fMRI: Functional magnetic resonance imaging
MRA: Magnetic resonance angiography
MRI: Magnetic resonance imaging
MS: Multiple sclerosis
RF: Radiofrequency
SNR: Signal-to-noise ratio
SWI: Susceptibility-weighted imaging
UHF: Ultra-high field

## References

[1] Yacoub E, Shmuel A, Pfeuffer J, van de Moortele PF, Adriany G, Andersen P, Vaughan JT, Merkle H, Ugurbil K, Hu X (2001). Imaging brain function in humans at 7 tesla. Magn Reson Med.

[2] Ultra-high field MRI scanners. https://www.google.com/maps/d/viewer. Accessed 10 June 2021

[3] Budinger TF, Bird MD (2018). MRI and MRS of the human brain at magnetic fields of 14 T to 20 T: technical feasibility, safety, and neuroscience horizons. Neuroimage.

[4] Siemens Healthineers. With 7 Tesla scanner Magnetom Terra, Siemens Healthineers introduces new clinical field strength in MR imaging. https://www.siemens.com/press/en/pressrelease/?press=/en/pressrelease/2017/healthineers/pr2017080391hcen.htm&content[]=HC2017. Accessed 10 June 2021

[5] FDA clears first 7T magnetic resonance imaging device 2017. https://www.fda.gov/NewsEvents/Newsroom/PressAnnouncements/ucm580154.htm. Accessed 10 June 2021

[6] Bringing ultra-high field MR imaging from research to clinical: SIGNA 7.0T FDA cleared https://www.ge.com/news/press-releases/bringing-ultra-high-field-mr-imaging-from-research-to-clinical-signa-70t-fda-cleared. Accessed 10 June 2021

[7] Ladd ME, Bachert P, Meyerspeer M, Moser E, Nagel AM, Norris DG, Schmitter S, Speck O, Straub S, Zaiss M (2018). Pros and cons of ultra-high-field MRI/MRS for human application. Prog Nucl Magn Reson Spectrosc.

[8] Dumoulin SO, Fracasso A, van der Zwaag W, Siero JCW, Petridou N (2018). Ultra-high field MRI: advancing systems neuroscience towards mesoscopic human brain function. Neuroimage.

[9] Sclocco R, Beissner F, Bianciardi M, Polimeni JR, Napadow V (2018). Challenges and opportunities for brainstem neuroimaging with ultrahigh field MRI. Neuroimage.

[10] Vachha B, Huang SY (2021) MRI with ultrahigh field strength and high performance gradients: challenges and opportunities for clinical neuroimaging at 7T and beyond. Eur Radiol Exp. 10.1186/s41747-021-00216-2 10.1186/s41747-021-00216-2 PMC8387544 34435246

[11] Webb AG, Collins CM (2010). Parallel transmit and receive technology in high-field magnetic resonance neuroimaging. Int J Imaging Syst Technol.

[12] Wiesinger F, Boesiger P, Pruessmann KP (2004). Electrodynamics and ultimate SNR in parallel MR imaging. Magn Reson Med.

[13] Ziegelberger G, Croft R, Feychting M (2020). Guidelines for limiting exposure to electromagnetic fields (100 kHz to 300 GHz). Health Phys.

[14] Tiberi G, Costagli M, Biagi L, Ciantis AD, Fontana N, Stara R, Symms MR, Cosottini M, Guerrini R, Tosetti M (2016). SAR prediction in adults and children by combining measured B1+ maps and simulations at 7.0 Tesla. J Magn Reson Imaging.

[15] Wald LL, Setsompop K (2012). Method for reducing local specific absorption rate in magnetic resonance imaging using radiofrequency coil array dark modes, April 23 2012. US Patent App. 13/453,208.

[16] Rauschenberg J, Nagel AM, Ladd SC, Theysohn JM, Ladd ME, Möller HE, Trampel R, Turner R, Pohmann R, Scheffler K, Brechmann A, Stadler J, Felder J, Shah NJ, Semmler W (2014). Multicenter study of subjective acceptance during magnetic resonance imaging at 7 and 9.4 T. Invest Radiol.

[17] Dula AN, Virostko J, Shellock FG (2014). Assessment of MRI issues at 7 T for 28 implants and other objects. AJR Am J Roentgenol.

[18] Fagan AJ, Bitz AK, Björkman-Burtscher IM, Collins CM, Kimbrell V, Raaijmakers AJE, ISMRM Safety Committee (2021). 7T MR safety. J Magn Reson Imaging.

[19] Bruschi N, Boffa G, Inglese M (2020). Ultra-high-field 7-T MRI in multiple sclerosis and other demyelinating diseases: from pathology to clinical practice. Eur Radiol.

[20] Absinta M, Sati P, Reich DS (2016). Advanced MRI and staging of multiple sclerosis lesions. Nat Rev Neurol.

[21] Cocozza S, Cosottini M, Signori A, Fleysher L, el Mendili MM, Lublin F, Inglese M, Roccatagliata L (2020). A clinically feasible 7-Tesla protocol for the identification of cortical lesions in multiple sclerosis. Eur Radiol.

[22] Maggi P, Absinta M, Grammatico M, Vuolo L, Emmi G, Carlucci G, Spagni G, Barilaro A, Repice AM, Emmi L, Prisco D, Martinelli V, Scotti R, Sadeghi N, Perrotta G, Sati P, Dachy B, Reich DS, Filippi M, Massacesi L (2018). Central vein sign differentiates multiple sclerosis from central nervous system inflammatory vasculopathies. Ann Neurol.

[23] Wang I, Oh S, Blümcke I, Coras R, Krishnan B, Kim S, McBride A, Grinenko O, Lin Y, Overmyer M, Aung TT, Lowe M, Larvie M, Alexopoulos AV, Bingaman W, Gonzalez-Martinez JA, Najm I, Jones SE (2020). Value of 7T MRI and post-processing in patients with nonlesional 3T MRI undergoing epilepsy presurgical evaluation. Epilepsia.

[24] Opheim G, van der Kolk A, Bloch KM, Colon AJ, Davis KA, Henry TR, Jansen JFA, Jones SE, Pan JW, Rössler K, Stein JM, Strandberg MC, Trattnig S, van de Moortele PF, Isabel Vargas M, Wang I, Bartolomei F, Bernasconi N, Bernasconi A, Bernhardt B, Björkman-Burtscher I, Cosottini M, Das SR, Hertz-Pannier L, Inati S, Jurkiewicz MT, Khan AR, Liang S, Ma RE, Mukundan S, Pardoe H, Pinborg LH, Polimeni JR, Ranjeva JP, Steijvers E, Stufflebeam S, Veersema TJ, Vignaud A, Voets N, Vulliemoz S, Wiggins CJ, Xue R, Guerrini R, Guye M (2021). 7T Epilepsy task force consensus recommendations on the use of 7T MRI in clinical practice. Neurology.

[25] Düzel E, Costagli M, Donatelli G, Speck O, Cosottini M (2021) Studying Alzheimer disease, Parkinson disease, and amyotrophic lateral sclerosis with 7-T magnetic resonance. Eur Radiol Exp. 10.1186/s41747-021-00221-5 10.1186/s41747-021-00221-5 PMC8387546 34435242

[26] Lehéricy S, Bardinet E, Poupon C, Vidailhet M, François C (2014). 7 tesla magnetic resonance imaging: a closer look at substantia nigra anatomy in Parkinson’s disease. Mov Disord.

[27] Cho ZH, Oh SH, Kim JM, Park SY, Kwon DH, Jeong HJ, Kim YB, Chi JG, Park CW, Huston J, Lee KH, Jeon BS (2011). Direct visualization of Parkinson’s disease by in vivo human brain imaging using 7.0T magnetic resonance imaging. Mov Disord.

[28] Cho ZH, Min HK, Oh SH, Han JY, Park CW, Chi JG, Kim YB, Paek SH, Lozano AM, Lee KH (2010). Direct visualization of deep brain stimulation targets in Parkinson disease with the use of 7-tesla magnetic resonance imaging: clinical article. J Neurosurg.

[29] van Laar PJ, Oterdoom DLM, ter Horst GJ, van Hulzen ALJ, de Graaf EKL, Hoogduin H, Meiners LC, van Dijk JMC (2016). Surgical accuracy of 3-tesla versus 7-tesla magnetic resonance imaging in deep brain stimulation for Parkinson disease. World Neurosurg.

[30] Cosottini M, Donatelli G, Costagli M, Caldarazzo Ienco E, Frosini D, Pesaresi I, Biagi L, Siciliano G, Tosetti M (2016). High-resolution 7T MR imaging of the motor cortex in amyotrophic lateral sclerosis. AJNR Am J Neuroradiol.

[31] Wisse LEM, Kuijf HJ, Honingh AM, Wang H, Pluta JB, Das SR, Wolk DA, Zwanenburg JJM, Yushkevich PA, Geerlings MI (2016). Automated hippocampal subfield segmentation at 7T MRI. AJNR Am J Neuroradiol.

[32] Solano-Castiella E, Schäfer A, Reimer E, Türke E, Pröger T, Lohmann G, Trampel R, Turner R (2011). Parcellation of human amygdala in vivo using ultra high field structural MRI. Neuroimage.

[33] Rotta J, Perosa V, Yakupov R, Kuijf HJ, Schreiber F, Dobisch L, Oltmer J, Assmann A, Speck O, Heinze HJ, Acosta-Cabronero J, Duzel E, Schreiber S (2021). Detection of cerebral microbleeds with venous connection at 7-tesla MRI. Neurology.

[34] De Rotte AAJ, Koning W, Den Hartog AG (2014). 7.0 T MRI detection of cerebral microinfarcts in patients with a symptomatic high-grade carotid artery stenosis. J Cereb Blood Flow Metab.

[35] Harteveld AA, De Cocker LJL, Dieleman N (2015). High-resolution postcontrast time-of-flight MR angiography of intracranial perforators at 7.0 tesla. PLoS One.

[36] Harteveld AA, van der Kolk AG, van der Worp HB, Dieleman N, Siero JCW, Kuijf HJ, Frijns CJM, Luijten PR, Zwanenburg JJM, Hendrikse J (2017). High-resolution intracranial vessel wall MRI in an elderly asymptomatic population: comparison of 3T and 7T. Eur Radiol.

[37] Sato T, Matsushige T, Chen B, Gembruch O, Dammann P, Jabbarli R, Forsting M, Junker A, Maderwald S, Quick HH, Ladd ME, Sure U, Wrede KH (2019). Wall contrast enhancement of thrombosed intracranial aneurysms at 7T MRI. AJNR Am J Neuroradiol.

[38] Harteveld AA, Van Der Kolk AG, Zwanenburg JJM (2016). 7-T MRI in cerebrovascular diseases: challenges to overcome and initial results. Top Magn Reson Imaging.

[39] Rutland JW, Delman BN, Gill CM, Zhu C, Shrivastava RK, Balchandani P (2020). Emerging use of ultra-high-field 7T MRI in the study of intracranial vascularity: state of the field and future directions. AJNR Am J Neuroradiol.

[40] Ross JS (2004). The high-field-strength curmudgeon. AJNR Am J Neuroradiol.

[41] Tanenbaum LN (2004). 3-T MR imaging: ready for clinical practice. AJNR Am J Neuroradiol.

[42] Shapiro MD, Magee T, Williams D, Ramnath R, Ross JS (2004). The time for 3T clinical imaging is now. AJNR Am J Neuroradiol.

